# The Role of Oncogenic Viruses in the Pathogenesis of Sporadic Breast Cancer: A Comprehensive Review of the Current Literature

**DOI:** 10.3390/pathogens13060451

**Published:** 2024-05-25

**Authors:** Chiara Rossi, Frediano Socrate Inzani, Stefania Cesari, Gianpiero Rizzo, Marco Paulli, Paolo Pedrazzoli, Angioletta Lasagna, Marco Lucioni

**Affiliations:** 1 Section of Anatomic Pathology, Cerba HealthCare Lombardia, 20139 Milan, Italy; 2 Department of Molecular Medicine, Unit of Anatomic Pathology, University of Pavia, IRCCS San Matteo Hospital Foundation, 27100 Pavia, Italy; 3 Unit of Medical Oncology, IRCCS San Matteo Hospital Foundation, 27100 Pavia, Italy

**Keywords:** breast cancer, carcinogenesis, viral carcinogenesis, HPV, MMTV, EBV, CMV, JCV, MCPYV

## Abstract

Breast cancer is the most common malignancy in the female sex; although recent therapies have significantly changed the natural history of this cancer, it remains a significant challenge. In the past decade, evidence has been put forward that some oncogenic viruses may play a role in the development of sporadic breast cancer; however, data are scattered and mostly reported as sparse case series or small case–control studies. In this review, we organize and report current evidence regarding the role of high-risk human papillomavirus, mouse mammary tumor virus, Epstein–Barr virus, cytomegalovirus, bovine leukemia virus, human polyomavirus 2, and Merkel cell polyomavirus in the pathogenesis of breast cancer.

## 1. Introduction

Since the first discovery of Rous sarcoma virus (RSV) and mouse mammary tumor virus (MMTV) as causative agents of cancer in animals in 1911 [1] and 1936 [2], the role of infectious agents as etiological agents of cancer has been studied and confirmed for several entities. In 2018, an analysis of the GLOBOCAN database reported that 13% of cancer was associated with infections, with *Helicobacter pylori*, human papillomavirus (HPV), hepatitis B virus (HBV) and hepatitis C (HCV) virus reported as the primary causative agents [3].

Viruses are recognized to cause or contribute to several human cancers; the list includes hepatocellular carcinoma (HBV and HCV), cervical squamous cell carcinoma and adenocarcinoma, head and neck squamous cell carcinoma (HPV), Burkitt’s lymphoma, nasopharyngeal carcinoma and a subset of gastric cancer [Epstein–Barr virus (EBV)], adult T-cell leukemia [human T-cell leukemia virus (HTLV1)], Kaposi’s sarcoma [human herpesvirus 8 (HHV8)], and Merkel cell carcinoma [Merkel cell polyomavirus (MCPyV)].

Viruses can participate in oncogenesis by different means, such as direct transcription of oncogenic proteins, by contributing to a chronic inflammatory milieu, and by causing genomic alterations and instability in the infected cells.

With cancer recognized as a multifactorial disease, any external factor that can be identified and acted upon, as primary or secondary prevention, becomes important to reduce cancer risk.

Since their introduction, Bradford Hill’s criteria [4] have been used to assess causality in epidemiology; there are nine items in those criteria: strength of association, consistency of association, specificity, temporality, biological gradient (dose–response relationship), plausibility, coherence, experiment, and analogy.

Therefore, in order to establish if a virus can be considered a causative agent of cancer, epidemiological studies need to be performed on a large scale, to identify the virus in the cancer cells, and consistently reproduce the results across regions and populations.

Breast cancer is the most common cancer in women; in 2022, there were 2.3 million of women diagnosed with breast cancer [5], and a few risk factors are known: female sex, genetic predisposition, increasing age, obesity, alcohol and tobacco use, family history, radiation exposure, reproductive history, early menarche and late first pregnancy, and postmenopausal hormone therapy. However, half of breast cancer cases occur in women with no known risk factor, except from age and female sex, and it is therefore likely that some risk factors are still undiscovered.

In this context, there is much interest in the scientific community in identifying new risk factors, in the hope of significantly impacting such a common cancer.

The possibility that viruses may have a role in the development of breast cancer has been investigated for some years for several viral agents, and many conflicting results have been reported in the literature.

In this review, we collect and examine the hypothesized pathogenetic mechanisms of viral breast carcinogenesis regarding HPV, MMTV, EBV, human cytomegalovirus (CMV), bovine leukemia virus (BLV), human polyomavirus 2 (JCV), and Merkel cell polyomavirus (MCPyV), we report the prevalence of these virus in breast cancer specimens, and we systematically review the results of studies that also included controls in their design to provide an up-to-date account of the progress that has been made in this field of study in recent years.

## 2. Materials and Methods

We performed a systematic review of the studies including controls in their study design present in breast cancer literature about the prevalence of HPV, MMTV, EBV, CMV, BLV, JCV and MCPyV for the 15-year period 2008–2023 using the keywords *((hpv) OR (human papillomavirus)) AND ((breast cancer) OR (BC) OR (breast neoplasia)) AND (pathogenesis), ((cmv)or(cytomegalovirus)) AND ((breast cancer) OR (BC) OR (breast neoplasia)) AND (pathogenesis), ((ebv)or(epstein-barr virus)) AND ((breast cancer) OR (BC) OR (breast neoplasia)) AND (pathogenesis), ((mmtv)or(mouse mammary tumor virus)) AND ((breast cancer) OR (BC) OR (breast neoplasia)) AND (pathogenesis), ((blv)or(bovine leukemia virus)) AND ((breast cancer) OR (BC) OR (breast neoplasia)) AND (pathogenesis), ((jcv)or(human polyomavirus 2)) AND ((breast cancer) OR (BC) OR (breast neoplasia)) AND (pathogenesis), ((MCPyV)or(merkel cell polyomavirus)) AND ((breast cancer) OR (BC) OR (breast neoplasia)) AND (pathogenesis).*

**Figure 1:**
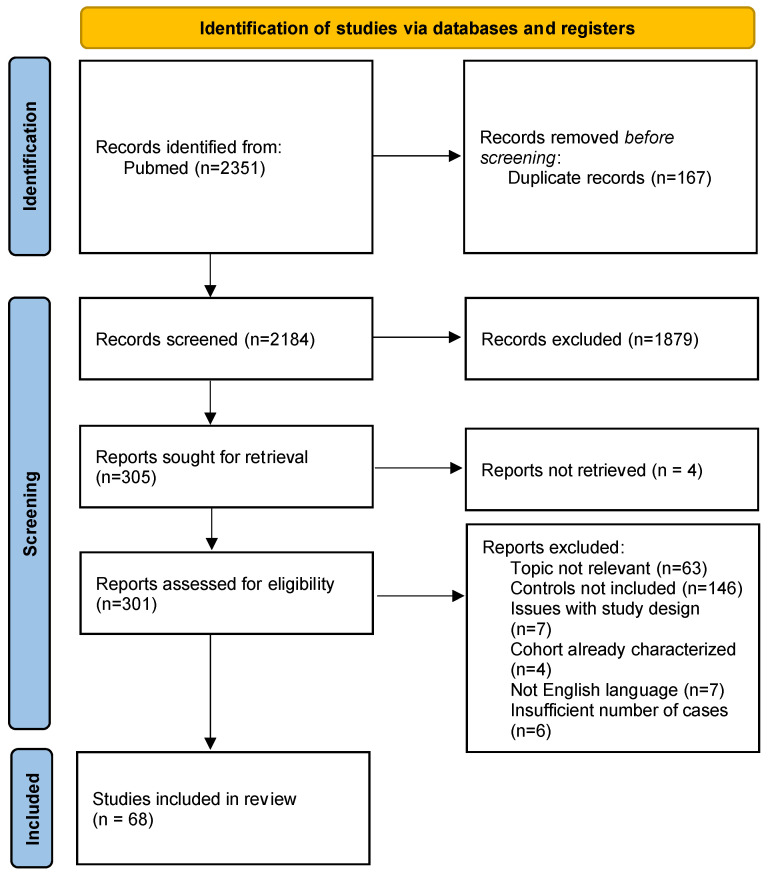
PRISMA 2020 flow diagram for systematic reviews.

A total of 2351 publications were screened according to the 2020 Prisma statement [6]; the results are summarized in Figure 1. Articles were imported into Microsoft Excel 2021 (https://www.microsoft.com/en-us/microsoft-365/excel, accessed on 1 May 2024) and duplicates were removed. Then, title and abstracts were screened to exclude a first group of publications not relevant to this work. Then, the full texts of the remaining articles were obtained, and the articles were further screened based on their content and relevance to the topic at hand. Exclusion criteria are detailed in Figure 1. A final count of 68 articles represented our cohort, and for that, we noted the author, year of publication, number of positive cases and positive controls, and types of controls. Fisher’s exact test and Pearson’s chi-square test (GraphPad Prism 5) were used to calculate the statistical significance (*p* ≤ 0.05 were considered significant) of case and control prevalence when appropriate, and they are reported in the tables in the text. Since most of the articles included in the systematic review were not structured like case–control studies, we refrained from calculating the odds ratio.

## 3. HPV

HPV is a family of DNA viruses that represent the most frequent sexually transmitted disease worldwide [7]. They are able to infect both skin and genital and oral mucosa, and they are responsible for a wide range of proliferative lesions, spanning from the common cutaneous wart to invasive cancer, most notably in the uterine cervix. They are categorized into low- and high-risk (HR) genotypes based on their association with cancer development. Most infections, even with HR genotypes, are asymptomatic, do not cause any visible lesion, and are cleared by the immune system in the months following the first infection; when it is not cleared, persistent infection is the most important risk factor for the development of HPV-linked cancer. The development of effective HPV vaccines has already changed the epidemiology of cervical cancer in countries where programs have been systematically implemented [8], and the effects will become more evident also for other types of cancer as the programs progress in time and become more inclusive.

HR-HPV is linked to the development of cervical, anogenital and head and neck carcinomas; it produces the oncogenic proteins E6 and E7, which have the potential to disrupt cell cycle by promoting degradation of the oncoprotein p53 and by binding the Rb protein, respectively.

HR-HPV relevance in the pathogenesis of breast cancer has been hypothesized after the discovery that HR-HPV can immortalize mammary epithelial cells in vitro [9,10], but an actual route of infection of the ductal epithelium in vivo has not been put forward yet.

There are three hypotheses for the mechanisms of infection [11]: through blood/lymphatics in a patient with previous HPV-positive gynecological cancer, by direct contact between genital and breast during sex through skin or nipple microabrasions, or by oral transmission to breast via bodily fluids. Another hypothesis for viral carcinogenesis is the so-called ‘hit and run’ mechanism, by which a virus may be involved in starting the carcinogenesis without integration into the host genome, and then subsequently cleared by the host immune defenses [12,13]. According to this hypothesis, the absence of measurable viral genetic material does not exclude its role in initiating the cancer process, and cancers that remain positive for the virus represent only a fraction of the whole; however, at the current time, there is not sufficient evidence to favor one hypothesis over the others.

Glenn et al. [14] also reported that HPV could be identified by polymerase chain reaction (PCR) in the milk of 6/40 (15%) women with no history of breast cancer; this mechanism of transmission was reported for MMTV, in which shedding of the virus in mother’s milk and lactation significantly increased the progeny’s risk of mammary cancer. A study by the European Institute of Oncology by Cazzaniga et al. [15] reported similar rates (14%) of positivity for HPV in ductal lavages, but the positivity was mostly confined to the nipple superficial epithelium, and the authors therefore surmised that it was a colonization or transient infection in the external epithelial cells of the nipple, and could not explain spread to the ductal epithelium of the breast.

Some authors reported koilocytosis in breast cancer specimens [16,17], suggesting a possible morphological clue for the link between HPV infection and breast cancer development. Some authors also investigated the possibility that an HPV infection at another site could increase the risk of breast cancer, through spread of the virus from the primary site to the breast via blood or lymphatics, as outlined above. On this note, it has been reported that women with a previous history of cervical intraepithelial neoplasia have a higher incidence of breast cancer [18,19].

Frega et al. [20] also investigated concurrent cervical HPV infection in the nine patients whose breast cancer had tested positive for HPV in their study population. They reported six (67%) cervical infections in the tested patients; in all cases, the patient shared at least one genotype between cervical and breast positivity. In the same study, Frega et al. also investigated nodal metastasis HPV positivity, and one case showed a positive HPV16 breast primary with a positive HPV16 nodal metastasis.

Data about HPV reported prevalence in breast cancer is reported in Table 1.

Reported prevalence for HPV ranged from 0% to 84% in the indexed literature in our study timeframe, with a median value of 18%, as reported in Table 1. Coinfection was frequently reported [10,20,22,26,31,35,43,44,52,57,60,64,68,72,81,83,86]; while coinfection has been found to be associated with higher stage and higher grade in cervical HPV-related lesions, no significant association has been reported in breast cancer in any of the studies reporting on it.

Variation in the reported prevalence can be due to a number of factors: choice of primer regions for PCR amplification, the sensibility of the techniques used for retrieval and amplification of viral DNA, source material (FFPE or CPT), storing conditions of the sample. Some of the studies employed a restricted set of HPV primers and only investigated HR genotypes, while others also screened for low-risk genotypes. Also, geographical variation of HPV distribution in the population is a known finding in cervical lesions, and it is also reflected in the diverse prevalence of HPV genotypes in different populations, and therefore may be reflected in a different prevalence also in breast cancer.

While it is difficult to compare the prevalence of specific HPV genotypes, due to the large variation of HPV primers, recent systematic reviews report the high-risk HPV16 as the most commonly detected HPV genotype in breast cancer [87,88]; other commonly reported genotypes are HPV18 and HPV33, both high-risk genotypes. From Table 1, our systematic review of the literature confirms HPV16, HPV18 and HPV33 as the most commonly detected HPV genotypes; however, we feel that these results are too dependent on the primer choice to be able to make strong inferences from them in this setting. Studies with a larger set of primers have also identified low-risk genotypes such as HPV6, whose relevance for breast carcinogenesis is unclear.

Choice of identification technique also has a significant impact on the results, and should be kept in mind when critically examining the results of the studies and comparing the reported incidence of HPV DNA in breast cancer [89]. Most of the studies use PCR to amplify the viral genome, a technique that is prone to contamination; it also makes it impossible, after amplification, to detect if the positivity for HPV comes from the breast cancer cells or from bystander stromal or immune cells. This is of particular relevance not only for HPV, but also for other viruses, such as EBV, that is often detected in lymphocytes.

When studies compared PCR rate of viral detection with in situ techniques, the reported positivity was lower in the in situ techniques rather than PCR [17,55]. Antonsson et al. [74] reported a 50% positivity for HPV when testing for the virus on CPT with PCR, but failed to report any positivity in tumor or adjacent breast tissue with ISH on FFPE. Frega et al. [20] reported nine positive cases on FFPE, but failed to demonstrate any viral mRNA positivity. Gannon et al. [56] sequenced the RNA transcript of 5 breast cancer samples that had tested positive for HPV DNA with PCR, as well as 53 available breast cancer and 10 normal breast tissue datasets, and did not identify any viral transcript.

This difference in the detection rate between PCR and ISH [55] has been explained by some authors as due to a low viral load in the ISH^−^/PCR^+^ cases; others consider this a possible spurious amplification of passerby or non-cancer related HPV genome by PCR. In general, HPV concentration in breast cancer seems to be lower than the concentrations detected in cervical cancer, making its detection more difficult [90].

Another important factor to keep in mind is that most of the studies reported only the detection of HPV DNA in the cancer tissue, without evaluating the presence of E6 and E7 transcripts, which is a hallmark of functional activity of the virus; therefore, we are not able to comment if the detected virus was actually able to influence the cellular milieu or was just an incidental finding.

Aguayo et al. [73] investigated both the integration and functionality status of HPV, and found the virus to be integrated and transcribed, albeit with a low viral load, but not functional.

Guo et al. [32], on the other hand, investigated the presence of HPV by chromogenic in situ hybridization (CISH), and only reported the CISH as positive when an integrative pattern was present; they therefore reported a 32% of breast cancer patients (both in situ and invasive) who were positive for integrated HPV DNA. In the same article, they also found HR-HPV to be significantly more amplified in the cancer than in the surrounding normal breast tissue [32]; no correlation with survival or clinico-pathological characteristics of the patients was found.

The study by Islam et al. [44] reported that 88% of HPV16 was present as integrated into the DNA of breast cancer. They also reported an increased number of HPV16 copies with increasing histological grade, stage and lymph node metastasis, and presence of the E6/E7 transcripts, indicating the function of the viral integration.

Khan et al. [86] reported the presence of integrated HPV DNA in all their 24 HPV16-positive cases; they did not report any instance of episomal DNA. Wang et al. [52] reported the presence of both episomal and integrated viral DNA in breast cancer; they did not, however, detect any significant viral transcription.

Due to the significant variation in conditions, choice of primers and population, it is not an easy task to compare results across the studies, also regarding the characteristics of breast cancer that different authors identified as possibly linked to HPV infection.

Most of the studies included cases that were a mix of invasive breast cancer histological types, with ductal carcinoma/carcinoma of no special type being the most commonly reported.

Some papers focused on specific histological types or molecular subtypes. Piana et al., Gupta et al. and Corbex et al. [31,61,64] investigated triple negative breast cancer in an Italian, a Croatian and an Algerian population. In their study, Piana et al. [64] reported a 15% vs. 0% of HPV positivity in triple negative vs. non-triple negative breast cancer. Corbex et al. [61] did report that triple negative breast cancer and inflammatory breast cancer had a stronger association to viral detection than other histological types, but did not link any specific virus to either triple negative or inflammatory breast cancer.

Herrera-Goepfert et al. [65] investigated HPV positivity rates in metaplastic breast cancer, and reported higher copy number than usually found in other histological types, while Duò et al. [85] performed PCR on 52 cases of breast cancer and found two positive results; both were invasive papillary carcinoma. Lastly, one case report by Kulka et al. [91] reported detection of HPV18 and HPV33 in a lymphoepithelioma-like carcinoma of the breast.

Islam et al. [44] reported worse survival in patients with breast cancer with positive HPV-DNA in tissue with respect to HPV-negative breast cancer patients, but it is the only study to report this association. Most studies [45,47,55,65,67,68,86] do not report any difference in age, tumor size, lymph node status or histological prognostic and predictive factors. De León et al. [81] reported a correlation with HPV DNA positivity and larger tumor size, but Antonsson et al. and Ghaffari et al. [74,75] reported the opposite, with HPV-positive tumors being smaller than HPV-negative counterparts, and having less nodal involvement.

Therefore, we do not consider HPV DNA detection in breast cancer to be associated with any difference in prognosis or tumor characteristics with respect to HPV-negative cancer.

To complete our review of the relevance of HPV-positivity in breast cancer, we analyzed the selected articles to evaluate the difference in prevalence between breast cancer and controls. The data are summarized in Table 2, as follows.

Pooling cases and controls together, there is an HPV prevalence of 24% (840/3450) in the cases and of 8% (158/1890) controls. *p*-value was significant (*p* < 0.0001).

However, it is not so straightforward to compare results across different papers, and therefore, the statistical power of this pooled analysis must also be taken with caution.

Study design between different papers is extremely heterogeneous, especially in regard to the control selection; in this systematic review, 7 control groups were fibroadenomas, 15 were unspecified benign lesions, 5 were normal breast tissue from the same patient, 8 were normal breast tissue from healthy patients, 4 were breast papillomas, 3 were fibrocystic disorder, and 6 studies included more than one of these categories. Chang et al. [70] reported HPV positivity in three benign phyllodes tumors. On the same topic of HPV positivity in fibroepithelial tumors, Alinezhadi et al. [23] also reported a 28% of positivity in their fibroadenoma control group, higher than the positivity in the control group; they also reported that this group had a lower incidence of HR-HPV genotypes with respect with the positives in the case group. While this may indicate that different HPV genotypes in breast may cause a spectrum of diseases, not only limited to epithelial malignancies or benign changes, it also raises issues with the inclusion of fibroepithelial tumors as negative controls in some of the case–control studies. Two studies from Gupta et al. [31] and Piana et al. [64] were excluded because the control group included malignant cases, to minimize the variability between control groups for the sake of this review.

The use of different controls did not impact the significance of the study. Since very different populations are used as controls in different papers, some variation would be expected; the fact that it is not observed is probably linked to a deficiency in statistical power from different studies, rather than an actual homogeneity.

Therefore, even if the pooled analysis is strongly significant, and this might imply some influence of HPV infection on breast cancer, we feel that the limitations in the design of the different studies detailed in this paragraph must be considered, and caution should be taken when analyzing the relevance of these data.

## 4. MMTV

Mouse mammary tumor virus (MMTV) is a betaretrovirus known to cause mammary cancer and lymphoma in mice, and has been identified also in breast milk. It was first discovered in 1936 by Bitter et al. [2] as a cancer-causing agent secreted in breast milk, and it sparked the notion that viruses can be the etiological agent of cancer. A human analogous virus, human mammary tumor virus (HMTV) or MMTV-like virus, has been proposed as a causative agent for human breast cancer [92].

However, in situ hybridization studies demonstrated that the detected DNA was homologous to human endogenous retroviruses [93] that represent vestigia from remote infections that were integrated into human DNA and have become inactivated by millennia of mutations. Moreover, the fact that there is no known cell receptor for MMTV in human cells also has not been explained or demonstrated in vivo, even if groups have reported transmission in in vitro cell cultures [94].

Correlation of the geographical incidence of breast cancer with the distribution of different mouse species in the early 2000s has been made [95], with *Mus domesticus*, the common house mouse, roughly paralleling the frequency of MMTV detection in breast cancer.

A study [96] by a group from University of Pisa reported the presence of MMTV DNA and RNA in saliva of children and adults, and in higher percentage in breast cancer patients, and proposed saliva as a route of interpersonal spread of infection. The presence of MMTV *env* genes have also been reported in 4% of human milk samples from healthy lactating women [97].

Data about MMTV reported prevalence in breast cancer is reported in Table 3.

Reported prevalence for MMTV ranged from 0% to 78% in the indexed literature in our study timeframe, with a median value of 15%, as reported in Table 3. As in the previous section, we excluded a paper by Cedro-Tanda et al. [116] because the cases included both benign and malignant lesions, and epithelial and fibroepithelial lesions; the decision was taken to keep the cohort of the review as homogeneous as possible as to minimize confounding factors.

One of the limits of interpretation of these data is that the only technique that is used for the detection of the virus is PCR, which is, as previously mentioned, a technique with several limits regarding localization of the virus positivity and extremely prone to contamination. Al Dossary et al. [103] actually reported a higher prevalence of MMTV-like *env* sequences in the adjacent normal tissue than in the cancer cells itself (10% vs. 6%). Perzova et al. [117] reported very low viral copy numbers in the analyzed samples, and deemed this result incompatible with a retrovirus-caused cancer; in their report, contamination from mice infesting the building was the most likely source of the unconvincing results.

One issue that has been raised by some authors as to explain the absence of viral transcripts in some of the experiments is the poor specificity of the PCR primer for the *env* sequence. However, the work by Fukuoka et al. [114] utilized the same primers as Pogo et al. [118] and still reported an absence of detection of virus in the 49 breast cancer cases sequenced.

Regarding integration of the virus in breast cancer cells, Lawson et al. [113] reported detection of the gp52 *env* protein by IHC in the nucleus of the cell; they claim this as proof of integration of the viral DNA into the cell after infection. It must be noted, however, that cross-reactivity has been documented in the literature [119,120].

Lawson et al. [113] also reported that the MMTV-positive breast cancer had ‘very similar’ histological features to the MMTV-positive mouse mammary tumors, with the description given in the paper resembling a medullary-like phenotype of human breast cancer. MMTV infection has been postulated to be linked to the development of pregnancy-associated breast cancer [121] and of inflammatory breast cancer [122], but not in a definitive manner.

Several authors [25,100,102,108,123] failed to report any statistical association between MMTV-like virus and clinicopathological characteristics of breast cancer. Hachana et al. [115] reported an inverse correlation between MMTV presence and progesterone receptor and HER2 status. De Sousa Pereira et al. [100] reported an increased frequency of MMTV-like *env* gene in HER2-enriched breast cancer, both of non-luminal and luminal histology. Naccarato et al. [101] found that MMTV *env*-like sequences were significantly more present in sporadic rather than hereditary breast cancer (30% vs. 4%). Mazzanti et al. [111] also evaluated the prevalence of MMTV in pre-invasive lesions and reported it in 27% of atypical ductal hyperplasia (ADH) and 82% of ductal carcinoma in situ.

To complete our review of the relevance of MMTV positivity in breast cancer, we analyzed the selected articles to evaluate the difference in prevalence between breast cancer and controls. The data are summarized in Table 4, as follows.

Pooling cases and controls together, there is an MMTV prevalence of 18% (189/1039) in the cases and of 5% (41/837) controls. *p*-value was significant (*p* < 0.0001).

Regarding the study design, there was a marked prevalence of normal breast tissue from the case group patients (7/11 studies) as control samples; while this is interesting regarding the restriction of detection of the virus to the tumor itself, it is not the common design of a case–control study, and therefore limits the strength of any consideration that could be made regarding epidemiological impact of MMTV infection.

For MMTV infection, the pooled analysis is strongly significant; however, the numbers and the design of the studies lack epidemiological power, even if some of the evidence might be compelling in suggesting a possible link.

## 5. EBV

EBV is a common herpesvirus in humans that infects B lymphocytes, and it is the etiological agent of infectious mononucleosis. It is estimated that close to 95% of adults have been infected worldwide, with infection rate peaking in late adolescence or earlier in developing countries. As with other herpesviruses, after the first infection, it is able to persist in the body in a latent stage, eventually recurring upon triggering. The primary route of transmission is oral, via the sharing of saliva of an infected individual, by infection of the epithelial cells of the tonsils; from there, the virus is able to infect B lymphocytes, and move to the germinal centers where the lymphocytes differentiate into memory B lymphocytes, where the virus reside in its latent phase [124,125]. It is strongly associated with nasopharyngeal and gastric cancer, endemic Burkitt’s lymphoma, post-transplant lymphoproliferative disorders and a proportion of Hodgkin’s lymphoma. Given the high prevalence of EBV antibodies in the normal population, and the fact that just a minority of people carrying a latent EBV infection develop cancer, establishing linkage and mechanisms of oncogenesis is not easy.

The possibility of a ‘hit and run’ mechanism (previously mentioned in the HPV section) has been suggested to explain a possible role of EBV in carcinogenesis for breast cancer. Some authors have reported that breast cancer cell lines infected with EBV have dysregulated HER2 and HER3 pathways [126]; other authors hypothesize that EBV infection may not be a causative agent, but rather infection during the course of the disease may alter the phenotype of the cancer, conferring a worse overall prognosis. Glenn et al. [14] reported that EBV could be identified by PCR in the milk of 14/40 (33%) women with no history of breast cancer.

Lorenzetti et al. [127] reported the detection in breast cancer cells of LMP2A, a gene associated with latency pattern II and III, that is reported in EBV-related neoplasms such as Hodgkin’s lymphoma and post-transplant lymphoproliferative disorders.

Data about EBV reported prevalence in breast cancer is reported in Table 5.

Reported prevalence for EBV ranged from 0% to 69% in the indexed literature in our study timeframe, with a median value of 23.5%, as reported in Table 5.

As for the previous viruses, some caveats must be made for the interpretation of the raw prevalence data reported: several studies that used in situ detection techniques actually reported the tumor-infiltrating lymphocytes (TILs) to be the source of the signal, rather than the cancer itself [151]; Khan et al. [151] reported a 48% positivity in their studies, but it was all confined to the tumor-infiltrating lymphocytes. In the study by Aguayo et al. [73], ISH for EBER1 showed that all the breast cancer cases were negative, while Corbex et al. [61] reported that only 1/10 PCR-detected cases showed EBER1 positivity in the cancer nuclei using ISH. Similar results were published by Baltzell et al. [144], that reported a 3% positivity by PCR in their cohort, but failed to confirm any positivity with ISH, and specified also that the PCR positivity had been localized in normal non-cancerous cells. Both Glaser et al. [152] and Hachana et al. [146] reported TILs as the only positive EBV localization and EBER-ISH source, respectively. Mekrazi et al. [129] also reported positivity in TILs rather than tumor cells when analyzing PCR-positive specimens with ISH.

Finally, Peng et al. [63] did report ISH positivity confined to the breast cancer cells, but did not report the number of cases tested with this technique and the percentage in which positivity was detected.

The discrepancy in ISH and IHC rate of positivity has been partially attributed to the quality of RNA material in FFPE specimens, which would contribute to the lower rate of detection by ISH; on this note, Glenn et al. [17] demonstrated a significant presence of EBV in breast cancer when using PCR on fresh frozen DNA extracts; however, when PCR was performed in situ in the same study, the percentage of positivity was actually higher in the control group rather than in the cancer group; the authors do not present an explanation for this finding. In contrast with this finding, Oliveira et al. [27] reported a 7% positivity for EBER1 with ISH, but found a 69% positivity for EBNA1 with immunohistochemistry.

No definitive association with prognostic and predictive factors was found [130,131,138] and the results at our disposal are contrasting: Hachana et al. [146] reported a correlation between EBV DNA presence and negativity for estrogen receptor; however, in the same article, they failed to demonstrate any positivity for EBV with ISH in tumor cells.

Oliveira et al. [27] found a higher prevalence of EBV in low grade and luminal A breast cancer, while both Corbex et al. [61] and Pai et al. [138] reported a higher prevalence of EBV in triple negative breast cancer; similarly, Mashaly et al. [153] reported an association with more aggressive tumors and an increased frequency of EBV subtype D in breast cancer patients; the same subtype was also associated with negativity for progesterone receptor and negativity for HER2. Also Mazouni et al. [148] reported EBV positive breast cancer to be more aggressive with high histological grade and a higher frequency of non-luminal phenotypes, while Zekri et al. [145] did not report any association with histological grade.

Finally, Ballard et al. [141] reported no significant preference for EBV detection in lobular breast cancer against ductal histology, which was instead suggested by Fawzy et al. [150], albeit with very small numbers in the lobular group (8 vs. 32 ductal carcinomas).

Zhang et al. [131] found that EBV positive breast cancer had a high positive expression for PD1/PDL1 and reported worse OS and DFS in EBV-positive patients. On the contrary, Marrão et al. and Joshi et al. [143,149] did not find any significant association between EBV positivity and viral load on OS; paradoxically, Marrão [143] reported that the latent form of EBV infection could confer a survival advantage to the patient. Analogous results were reported by Heng et al. [154] that utilized the Health of Women study database to investigate an association between infectious mononucleosis as a surrogate for EBV infections and breast cancer; the author reported that women diagnosed with infectious mononucleosis before 22 years of age had lower risk of breast cancer (adjusted odds ratio 0.83) than women that never contracted EBV.

El-Naby et al. and Shahi et al. [130,139] reported a higher nodal status in EBV-positive patients. Mostafaei et al. [135] reported higher levels of inflammatory markers such as TNFα in patients with EBV+ breast cancer than in controls.

To complete our review of the relevance of EBV positivity in breast cancer, we analyzed the selected articles to evaluate the difference in prevalence between breast cancer and controls. The data are summarized in Table 6, as follows.

Pooling cases and controls together, there is an EBV prevalence of 24% (290/1201) in the cases and of 5% (47/877) in the controls. *p*-value was significant (*p* < 0.0001).

Regarding the study design, there was a prevalence of mixed benign lesions (7/16 studies) as control samples. This makes any consideration that goes beyond ‘benign vs. malignant’ very complex, because we cannot make any inference about the contribution of EBV on the pathogenesis of other lesions other than invasive breast carcinoma; for example, the two papers by Sharifpour et al. [137] and El-Naby et al. [139] reported a 18% and 14% rate of positivity also in fibroadenomas.

Also, for EBV, as for the previously reported viruses, the pooled statistical significance of the cases and controls is very strong; however, as for the previously reported viruses, there are important limitations that must be taken into account for interpreting the data. The very frequent report that EBV positivity comes from TILs makes PCR-only studies unreliable.

## 6. CMV

Cytomegalovirus (CMV) is a frequent virus in humans; around 40 to 90% of the population will contract the infection during their lifetime. The viral cycle consists of alternate lytic and latent phases of infection, where the virus is kept under control by the immune system, and reactivates with inflammatory states or immunodepression; it usually presents with an asymptomatic course or with very mild symptoms in immunocompetent patients, but its reactivation can cause severe and life-threatening disease in immunocompromised hosts, such as pneumonitis, meningitis, colitis, and hepatitis.

The relationship of CMV with cancer is a complex and controversial one, but even if its exact role has not been clarified as of yet in any of the suggested malignancies [155], it has been investigated in relation to various important aspects of carcinogenesis, including tumor microenvironment, epithelial–mesenchymal transition, and overall immune system regulation [156]. It has also been shown to be able to trigger oncogenic transformation in mammary epithelial cells in vitro [157,158] via the development of polyploid giant cells, with downstream activation of the oncogene c-Myc and EZH2 [159,160]. These strains have been referred to as high-risk CMV, referencing the common terminology used for HPV; the same French group was also able to report the isolation of two of these high-risk CMV strains from two triple negative breast cancer biopsies [161]. Kumar et al. [157] reported that infected cells were capable of causing mammary tumors in transfected mice.

Data about CMV reported prevalence in breast cancer is reported in Table 7.

Reported prevalence for CMV ranged from 0% to 100% in the indexed literature in our study timeframe, with a median value of 22%, as reported in Table 7.

The heterogeneity of techniques used for virus detection is, as previously mentioned, a strong limit in the reproducibility and correct evaluation of the significance of the reported findings; for example, when confirming IHC results with qPCR, Touma et al. [164] were only able to detect viral DNA in 30% of the tested cases. Therefore, IHC only reports might be considered unreliable. The detection of CMV full genome was reported in some of the works by Herbein’s group [160], but not in other works of the same group, [157], which also makes selection of the PCR primer important and might explain poor detection rates in some of the studies, also depending on the phase in which the virus is detected inside the cell (lytic vs. latent).

El-Shinawi [168] reported a higher frequency of CMV positivity in inflammatory breast cancer than in any other histotype. They also reported an increased activation of NF-κB p65 in CMV-positive inflammatory breast cancer. On a similar note, Costa et al. [165] reported a positive association between extensive positivity for CMV IE protein and the proinflammatory mediators COX-2 and 5-LO.

In their paper, Nakhaie et al. [163] reported not only a significant difference in CMV detection rate between breast cancer tissue and adjacent normal tissue, but also a significant difference in the detection of anti-CMV IgG, which were statistically higher in the breast cancer patients, possibly reinforcing the hypothesis that late exposure to the virus may be linked to the development of breast cancer. However, Pandey et al. [169] reported a contrary result in a larger cohort, in which IgG against CMV glycoprotein-B were higher in the healthy control population than in breast cancer patients.

Touma et al. [162] reported a shorter overall survival (OS) for patients who were positive for CMV IE protein at diagnosis; however, no information about treatment, comorbidity or tumor biology and stage was included in this analysis. Using a regression analysis, only expression of LA protein showed a tendency toward significance for a shorter OS, but that also failed to reach significance.

No association of CMV positivity and lymph node status, tumor dimension or proliferation index was reported [165,167].

To complete our review of the relevance of CMV positivity in breast cancer, we analyzed the selected articles to evaluate the difference in prevalence between breast cancer and controls. The data are summarized in Table 8, as follows.

Pooling cases and controls together, there is a CMV prevalence of 12% (50/416) in the cases and of 5% (16/350) controls. *p*-value was significant (*p* = 0.0003). However, despite this significant statistical result, we feel that the numbers considered are actually too small to draw strong epidemiological inferences.

## 7. BLV

Bovine leukemia virus (BLV) is a bovine oncogenic retrovirus that causes a disease similar to B cell leukemia. The presence in humans of antibodies that react to BLV antigens supports the theory that a zoonotic transmission of the virus may happen via direct contact or consumption of contaminated products, but a clear transmission mechanism has not been clearly demonstrated, nor has its implication as a causative cancer agent. Moreover, results regarding the actual prevalence of BLV antibodies in serum samples are controversial, ranging from 0% to 74.3% in a recent metanalysis [170]. Some authors believe transmission from cattle to human to be plausible, as the bovine infection is widespread; consumption of raw, unpasteurized milk and raw or undercooked beef might be the vessel, as the virus is rendered non-infectious by heat.

Data about BLV reported prevalence in breast cancer is reported in Table 9.

Reported prevalence for BLV ranged from 0% to 96% in the indexed literature in our study timeframe, with a median value of 37.5%, as reported in Table 9. As previously reported in other sections of this review, a study by Khan et al. [182] was excluded because the case group included benign, malignant and infectious lesions.

As with most of the previously mentioned viruses, we briefly touch again on the topic of PCR limitations, because we feel that it is an important factor to properly analyze the results; in this case, we stress once again the heterogeneous results that primer choice can make; for example, the gene regions *gag*, *pol* and *env* are often eliminated from the DNA as the tumor progresses, while the *tax* region is conserved longer; this is nicely highlighted by the results by Delarmelina et al. [175], which reported a 94% positivity when using a primer annealing the *tax* gene, which dropped to 57% when using a primer against the *env* gene.

Buehring et al. [180] analyzed the prevalence of BLV DNA across a biological gradient of lesions; they included patients with ‘premalignant’ conditions, such as ADH and carcinoma in situ, and reported that 38% of tissues with these lesions were positive for BLV DNA (against 59% of malignant tissues, and 29% of tissues from healthy controls).

Interestingly, Buehring et al. [177] also investigated if a temporal relationship between exposure and cancer development exists; they analyzed 48 subjects, for which a benign breast sample from previous surgery was present. Of these 48 subjects, 28 (58%) developed cancer. Of the patients that developed cancer, 20/28 had BLV DNA detectable in both specimens, and in 3 patients that had a premalignant diagnosis in the first specimen, BLV DNA was detectable both in the premalignant and in the malignant specimen.

No association between BLV DNA detection in breast cancer and predictive and prognostic factors was reported by any of the authors [174,175,176,180].

To complete our review of the relevance of BLV positivity in breast cancer, we analyzed the selected articles to evaluate the difference in prevalence between breast cancer and controls. The data are summarized in Table 10, as follows.

Pooling cases and controls together, there is a BLV prevalence of 57% (238/421) in the cases and of 30% (133/447) controls. *p*-value was significant (*p* < 0.0001). However, as for CMV in the previous paragraph, we feel that there are not large enough samples to draw strong epidemiological conclusions.

## 8. JCV

JCV, or human polyomavirus 2, is a polyomavirus whose asymptomatic infection is detected in 80–90% of immunocompetent adults, and it has been shown to cause progressive multifocal leukoencephalopathy and other disease in immunocompromised patients only. Sequences of the virus have been detected across several neoplasias [183,184,185,186], but the results are controversial and mostly inconclusive.

Two studies investigated the prevalence of JCV in breast cancer.

One study by Dowran et al. [136] failed to detect any virus DNA by PCR in 150 cases and 150 controls. On the other hand, Hachana et al. [187] reported a 23% (28/123) prevalence of JCV in a Tunisian breast cancer cohort, and suggested a correlation to a triple negative phenotype.

The data in the literature do not appear to support a possible contribution of JCV in breast cancer.

## 9. MCPyV

Merkel cell polyomavirus (MCPyV) is a human polyomavirus, thought to be the causative agent of the majority of cases of Merkel cell carcinoma, a highly aggressive neuroectodermal skin cancer. It appears to be a rather common infection in the general population, that can be detected on healthy skin in 60–90% of various sites, where it infects dermal fibroblasts and persists in a latent, episomal form of the viral genome [188]; infection occurs early in life, through shedding of the virions from healthy skin, either via skin infection or via the respiratory tract.

Data about MCPyV reported prevalence in breast cancer is reported in Table 11.

Reported prevalence for MCPyV ranged from 0% to 14% in the indexed literature in our study timeframe, with a median value of 2%, as reported in Table 11.

One study by Peng et al. [63] investigated the prevalence of MCPyV also in healthy controls and reported a positivity rate of 2% (1/50) vs. 14% in neoplastic cases (*p* value = 0.0210); similar values were also reported by Reza et al. [106] (3% detection rate in cases, 0% detection in the controls).

The data in the literature do not appear to support a possible contribution of MCPyV in breast cancer.

## 10. Conclusions

To our knowledge, this review represents the most up-to-date work on the topic at hand. While we recognize that it has limitations, such as the heterogeneity of the papers included, that rendered a systematic meta-analysis impossible, and the fact that we only included results from Pubmed, we think that the complete overview that it provides is still of value.

As per our judgment, no evidence appears to exist to suggest that JCV or MCPyV may contribute to the development of breast cancer; for CMV and BLV we feel that, even if some interesting data exist regarding prevalence of infection, case–control studies are too limited to draw strong conclusions.

Regarding the contribution of HPV, MMTV and EBV to breast carcinogenesis, extensive raw data exist in the literature that might support their involvement, but contribution is severely hindered by the small cohorts analyzed, the variety of techniques and samples that are included, and the lack of scientifically rigorous study design. While some evidence might be compelling, and could have an important impact on public health strategies, we advocate for the need of extensive and robust epidemiological studies to advance the knowledge in this field. A more uniform choice of test, with a preference for in situ techniques that allow for a correct visualization of the positivity signal, could improve the confidence in positive results, as well as the design of proper case–control studies on a larger scale; for HPV, we also feel that the introduction of large-scale vaccination campaigns and their possible effect on breast cancer prevention as well should be properly investigated in the next few years.

## Figures and Tables

**Table 1:** Prevalence of HPV DNA detection in breast cancer samples according to different studies.

Study	Positive Cases/Total Cases (%)	Genotypes Detected (Detected/Cases)	Technique Used
Haghighi, 2023 [21]	23/90 (25%)	HPV16 (10/23), HPV18 (9/23), HPV33 (2/23).	RT-PCR on CPT
Maroga, 2023 [22]	78/101 (77%)	HPV16 (37/101) HPV70 (36/101), HPV51 (22/101), HPV35 (16/101), HPV82 (16/101), HPV18 (5/101).	PCR on FFPE sections
Alinezhadi, 2022 [23]	8/63 (13%)	HPV33 (3/8), HPV16 (2/8), HPV11 (1/8).	PCR on FFPE sections
Calderon, 2022 [24]	13/447 (3%)	HPV16 (11/13), HPV18 (2/13).	PCR on CPT (controls) and FFPE sections (cases)
Gupta, 2022 [25]	48/74 (65%)	//	PCR on FFPE TMA
Mamani-Zapana, 2022 [26]	27/32 (84%)	HPV18 (19/27), HPV16 (8/27).	RT-PCR on FFPE sections
Oliveira, 2022 [27]	0/54 (0%)	//	Nested-PCR on FFPE sections
Charostad, 2021 [28]	12/36 (33%)	HPV16 (8/12), HPV18 (2/12), HPV31 (1/12), HPV6 (1/12).	PCR on CPT
Elagali, 2021 [29]	13/150 (9%)	HPV16 (5/13), HPV58 (4/13), HPV18 (3/13), HPV11 (1/13).	PCR on FFPE sections
Golrokh Mofrad, 2021 [30]	7/59 (12%)	HPV18 (5/7), HPV6 (2/7).	PCR on FFPE sections
Gupta, 2021 [31]	37/70 (53%)	HPV52 (18%), HPV45 (11%), HPV31 (4%), HPV58 (4%), HPV68 (1%).	PCR on FFPE sections
Guo, 2021 [32]	58/180 (32%)	HPV16/18 (39/58), HPV6/11 (19/58).	CISH on FFPE sections
Sher, 2020 [33]	5/50 (10%)	HPV16 (3/5), HPV35 (3/5), HPV58 (1/5).	PCR on fresh breast tissue
Tawfeik, 2020 [34]	4/20 (20%)	HPV16 (3/4), HPV18 (1/4).	RT-PCR on CPT
Balci, 2019 [35]	8/18 (44%)	HPV11 (8/8), HPV39 (2/8), HPV6 (1/8), HPV82 (1/8)	PCR on FFPE sections
Baltzell, 2018 [36]	2/61 (3%) by IS-PCR 4/61 (7%) by ISH	HPV16	IS-PCR and ISH on FFPE sections
Cavalcante, 2018 [37]	51/103 (50%)	HPVX (40/51), HPV6/11 (7/51), HPV18 (2/51), HPV33 (2/51)	Nested-PCR on FFPE samples
Ghaffari, 2018 [38]	4/72 (6%)	//	Nested-PCR on FFPE sections
Habyarimana, 2018 [39]	22/47 (47%)	HPV16 (77%), HPV33(14%), HPV31 (9%)	Nested-PCR on FFPE sections
Kouloura, 2018 [40]	0/201 (0%)	//	E1-based PCR on TMA
Malekpour Afshar, 2018 [41]	8/98 (8%)	HPV16 (5/8), HPV18 (5/8), HPV31 (3/8), HPV33 (3/8)	rtPCR on FFPE
Bakhtiyrizadeh, 2017 [42]	0/150 (0%)	//	PCR on FFPE
Delgado-García, 2017 [43]	130/251 (52%)	//	PCR on FFPE sections
Islam, 2017 [44]	174/272 (64%)	HPV16 (120/174), HPV18 (61/174) and HPV33 (5/174).	PCR and IHC on fresh tissue
Naushad, 2017 [45]	45/250 (18%)		PCR on FFPE sections
Salman, 2017 [46]	35/74 (47%)	HPV39 (13/35), HPV18 (8/35), HPV45 (8/35), HPV16 (7/35), HPV35 (7/35), HPV59 (7/35).	type-specific PCR on fresh tissue
Wang, 2017 [47]	14/81 (17%)	//	hybrid capture 2 on fresh tissue
Wang, 2017 [48]	14/50 (28%)	HPV16 (14/14)	qRT-PCR on FFPE
Doosti, 2016 [49]	20/87 (23%)	HPV6 (9/20), HPV16 (7/20), HPV18 (3/20), HPV11 (1/20).	Nested-PCR on FFPE
Ilahi, 2016 [50]	8/46 (17%)	HPV16 (8/8)	PCR on FFPE sections
Yan, 2016 [51]	23/76 (30%)	HPV18 (23/23)	Dual-PCR
Wang, 2016 [52]	52/146 (36%)		ISH on FFPE sections
Li, 2015 [53]	3/187 (2%)	HPV6 (1/3), HPV18 (1/3), HPV16 (1/3).	PCR on FFPE
Fimereli, 2015 [54]	2/58 (3%) with PCR 0/58 (0%) with transcriptome	HPV16 (1/2), HPV-HR (1/2)	RNA sequencing, exome sequencing PCR and IHC on CPT and FFPE sections
Fu, 2015 [55]	25/169 (15%) with PCR 17/169 (10%) with ISH	HPV58 (25/25)	PCR and ISH on FFPE
Gannon, 2015 [56]	13/80 (16%)		Nested-PCR on CPT and RNA sequencing
Salehpour, 2015 [57]	54/206 (26%)	HPV11 (19/54), HPV6 (5/54), HPVX (48/54).	PCR on FFPE sections
Vernet-Tomas, 2015 [58]	0/76 (0%)	//	PCR on FFPE sections
Ahangar-Oskouee, 2014 [59]	22/65 (34%)	HPV6 (17/22), HPV11 (1/22), HPV16 (1/22), HPV35 (1/22), HPV52 (1/22).	Nested-PCR on FFPE
Ali, 2014 [60]	60/129 (47%)	HPV31 (39/129), HPV18 (35/129), HPV16 (33/129), HPV33 (16/129).	ISH on FFPE sections
Corbex, 2014 [61]	15/123 (12%)	HPV16 (8/15), HPV31 (3/15), HPV22 (2/15).	PCR on FFPE sections
Manzouri, 2014 [62]	10/55 (18%)	HPV12 (2/10), HPV11 (2/10), HPV8 (1/10), HPV33 (1/10), HPV35 (1/10), HPV45 (1/10), HPV55 (1/10).	PCR on FFPE sections
Peng, 2014 [63]	2/100 (2%)	HPV18 (2/2)	Multiplex PCR detected by matrix-assisted laser desorption ionization-time of flight mass spectrometry on CPT
Piana, 2014 [64]	6/80 (8%)	HPV16 (28.6%), HPV31 (14.3%), HPV45 (14.3%), HPV52 (14.3%), HPV6 (14.3%), HPV66 (14.3%)	PCR on FFPE sections
Herrera-Goepfert, 2013 [65]	8/20 (40%)	HPV16 (7/8), HPV18 (1/8).	PCR on FFPE sections
Hossein, 2013 [66]	52/150 (35%)	HPV16 (21/52), HPV18 (15/52), HPV11 (8/52), HPV31 (4/52), HPV33 (2/52), HPV35 (2/52).	PCR on FFPE sections
Lieng, 2013 [67]	48/224 (21%)	//	hybrid capture 2 assay
Pereira Suarez, 2013 [68]	16/61 (26%)	HPV11 (4/16), HPV16 (3/16), HPV13 (1/16).	PCR on fresh tissue
Baltzell, 2012 [69]	2/70 (3%) with IS-PCR 4/70 (6%) with ISH	//	ISH and IS-PCR on FFPE sections
Chang, 2012 [70]	0/48 (0%)	//	FQ-PCR and ISH on FFPE sections
Frega, 2012 [20]	9/31 (29%)	HPV16 (44%), HPV6 (22%).	//
Glenn, 2012 [17]	25/50 (50%) with liquid PCR 10/27 (37%) with IS-PCR	HPV18 (25/25)	Liquid PCR on CPT, IS-PCR on FFPE sections
Herrera-Romano, 2012 [71]	0/118 (0%)	//	PCR on FFPE microdissected FFPE sections
Sigaroodi, 2012 [72]	15/79 (26%)	HPV18 (4/15), HPV16 (4/15), HPV6 (2/15), HPV23 (2/15) HPV124 (1/15), HPV15 (1/15), HPV11 (1/15).	PCR on FFPE sections
Aguayo, 2011 [73]	4/46 (9%)	HPV16 (4/4)	RT-PCR on FFPE sections
Antonsson, 2011 [74]	27/54 (50%)	HPV18 (27/27)	PCR on CPT and ISH on FFPE
Ghaffari, 2011 [75]	30% (out of 67; exact number not provided)	HPV16 (15%). HPV11/16 (12%), HPV31/33 (3%). Exact number not provided	PCR on CPT
Hedau, 2011 [76]	0/228 (0%)	//	qRT-PCR on CPT
Mou, 2011 [77]	4/62 (7%)	HPV16 (3/4), HPV18 (1/4)	PCR on CPT
Silva, 2011 [78]	0/79 (0%)	//	PCR on FFPE sections
Hachana, 2010 [79]	0/123 (0%)	//	PCR and ISH on FFPE sections
Yavuzer, 2010 [80]	0/84 (0%)	//	Nested-PCR on FFPE sections
De León, 2009 [81]	15/51 (29%)	HPV16 (11/15), HPV18 (4/15).	PCR on FFPE sections
He, 2009 [82]	24/40 (60%)	HPV16 (24/24).	PCR on CPT
Heng, 2009 [16]	8/26 (31%)	HPV18 (7/8), HPV16 (1/8).	IS-PCR on FFPE
Mendizabal-Ruiz, 2009 [83]	3/67 (4%)	HPV31 (2/3), HPV6 (1/3), HPV16 (1/3), HPV35 (1/3), HPV18 (1/3)	PCR on FFPE
Akil, 2008 [10]	69/113 (61%)	HPV33 (63/69), HPV35 (42/69), HPV18 (11/69), HPV16 (10/69), HPV31 (8/69)	PCR on FFPE TMA
de Cremoux, 2008 [84]	0/50 (0%)	//	PCR on unspecified material
Duò, 2008 [85]	2/52 (4%)	HPV66, HPVX	PCR on FFPE sections
Khan, 2008 [86]	26/124 (21%)	HPV16 (24/26), HPV6 in (12/26), HPV18 (3/26), HPV51 (2/26), HPV33 (1/26).	qRT-PCR on FFPE

RT-PCR, real-time PCR; CPT, cryopreserved tissue; //, data not available; FFPE, formalin-fixed, paraffin-embedded; TMA, tissue microarray; CISH, chromogenic in situ hybridization; IS-PCR, in situ PCR; qRT-PCR, quantitative real-time PCR; ISH, in situ hybridization; IHC, immunohistochemistry; FQ-PCR, fluorescence quantitative PCR.

**Table 2:** Summary of the prevalence of HPV detection of investigated cases and controls.

Study	HPV-Positive Cases/Total	HPV-Positive Controls/Total	Statistically Significant? Y/N (*p*-Value)
Haghighi, 2023 [21]	23/90 (25%)	7/32 (22%) ^^^	No (*p* = 0.8127)
Alinezhadi, 2022 [23]	8/63 (13%)	9/32 (28%) *	No (*p* = 0.0890)
Calderon, 2022 [24]	5/447 (3%)	1/79 (1%) ^#^	No (*p* = 1.0000)
Golrokh Mofrad, 2021 [30]	7/59 (12%)	0/11 (0%) ^#^	No (*p* = 0.5866)
Charostad, 2021 [28]	12/36 (33%)	2/36 (6%) ^§^	Yes (*p* = 0.0059)
Guo, 2021 [32]	58/180 (32%)	10/131 (8%) ^^,$^	Yes (*p* < 0.0001)
Sher, 2020 [33]	5/50 (10%)	8/100 (8%) *^,#,$^	No (*p* = 0.7607)
Tawfeik, 2020 [34]	4/20 (20%)	0/15 (0%) ^#^	No (*p* = 0.1186)
Baltzell, 2018 [36]	2/61 (3%) by IS-PCR 4/60 (7%) by ISH	8/100 (8%) ^§^ by IS-PCR 3/95 (3%) by ISH	No by IS-PCR (*p* = 0.3211) No by ISH (*p* = 0.4310)
Cavalcante, 2018 [37]	51/103 (50%)	15/95 (16%) ^^^	Yes (*p* < 0.0001)
Malekpour Afshar, 2018 [41]	8/98 (8%)	0/40 ^&^	No (*p* = 0.1046)
Bakhtiyrizadeh, 2017 [42]	0/150 (0%)	0/150 ^#^	//
Delgado-García, 2017 [43]	130/251 (52%)	49/186 (26%) ^#^	Yes (*p* < 0.0001)
Islam, 2017 [44]	174/272 (64%)	10/38 (26%) ^^,^*	Yes (*p* < 0.0001)
Salman, 2017 [46]	35/74 (47%)	11/36 (31%) ^^,#^	No (*p* = 0.1045)
Doosti, 2016 [49]	20/87 (23%)	0/84 ^&^	Yes (*p* < 0.0001)
Wang, 2016 [52]	52/146 (35%)	3/83 ^#^	Yes (*p* < 0.0001)
Li, 2015 [53]	3/187 (2%)	0/92 ^#^	No (*p* = 0.5532)
Gannon, 2015 [56]	13/80 (16%)	1/10 (10%) ^#^	No (*p* = 1.0000)
Ahangar-Oskouee, 2014 [59]	22/65 (34%)	0/65 *^,#,$^	Yes (*p* < 0.0001)
Ali, 2014 [60]	60/129 (47%)	0/20 (0%) ^^^	Yes (*p* < 0.0001)
Manzouri, 2014 [62]	10/55 (18%)	7/51 (14%) ^#^	No (*p* = 0.6029)
Peng, 2014 [63]	2/100 (2%)	0/100 ^#^	No (*p* = 0.4975)
Lieng, 2013 [67]	48/224 (21%)	6/37 (16%) *	No (*p* = 0.6611)
Chang, 2012 [70]	0/48 (0%)	3/30 (10%) ^#^	No (*p* = 0.0534)
Frega, 2012 [20]	9/31 (29%)	0/12 *^,$^	Yes (*p* = 0.0438)
Glenn, 2012 [17]	10/27 (37%)	3/18 (17%) ^^^	No (*p* = 0.1882)
Sigaroodi, 2012 [72]	15/79 (26%)	1/41 (2%) ^§^	Yes (*p* = 0.0105)
Mou, 2011 [77]	4/62 (7%)	0/46 (0%) ^§^	No (*p* = 0.1345)
de León, 2009 [81]	15/51 (29%)	0/43 (0%) *^,&^	Yes (*p* < 0.0001)
He, 2009 [82]	24/40 (60%)	1/20 (5%) ^^^	Yes (*p* < 0.0001)
Heng, 2009 [16]	8/26 (31%)	3/17 (18%) ^^^	No (*p* = 0.4801)
Mendizabal-Ruiz, 2009 [83]	3/67 (4%)	0/40 (0%) ^#^	No (*p* = 0.2911)

* fibroadenomas, ^#^ unspecified benign lesions, ^§^ normal breast tissue from the same patient, ^^^ normal breast tissue from healthy patients, ^$^ breast papillomas, ^&^ fibrocystic disorder, //, data not available.

**Table 3:** Prevalence of MMTV detection in breast cancer samples according to different studies.

Study	Positive Cases/Total Cases (%)	Technique Used
Fekete, 2023 [98]	0/75 (0%)	PCR for MMTV-like *env* gene on FFPE sections
Gupta, 2022 [25]	11/74 (15%)	PCR on FFPE sections
Gupta, 2021 [31]	5/70 (7%)	PCR on FFPE sections
Khalid, 2021 [99]	69/105 (65%)	qPCR on FFPE sections
De Sousa Pereira, 2020 [100]	41/217 (19%)	Nested-PCR for MMTV-like *env* gene on fresh tissue
Naccarato, 2019 [101]	19/103 (18%)	Fluorescence nested-PCR of microdissected FFPE sections for MMTV *env*-like sequences gene on fresh tissue
Seo, 2019 [102]	12/128 (9%)	PCR for HMTV on fresh tissue
Al Dossary, 2018 [103]	9/103 (9%)	Reverse transcriptase PCR for MMTV *env*-like sequences on FFPE sections
Motamedifar, 2017 [104]	0/50 (0%)	PCR for MMTV *env* genes on FFPE sections
Naushad, 2017 [45]	83/250 (29%)	PCR on FFPE sections
Shariatpanahi, 2017 [105]	19/59 (32%)	Nested-PCR for MMTV-like *env* sequences on FFPE sections
Reza, 2015 [106]	12/100 (12%)	RT-PCR of FFPE sections
Ahangar Oskouee, 2014 [107]	0/65 (0%)	Nested-PCR for MMTV-like sequences on FFPE sections
Naushad, 2014 [108]	16/80 (20%) for *env* gene 21/80 (26%) for LTR sequences	PCR for MMTV *env*-like and LTR sequences on FFPE sections
Morales-Sánchez, 2013 [109]	0/86 (0%)	PCR for *env* gene on CPT
Tabriz, 2013 [110]	0/40 (0%)	RT-PCR for MMTV-like *env* gene on FFPE sections
Glenn, 2012 [17]	39/50 (78%) with liquid PCR, 5/27 (19%) with IS-PCR	Liquid PCR on fresh frozen tissue, IS-PCR on FFPE sections
Mazzanti, 2011 [111]	7/20 (35%)	Fluorescence nested-PCR for MMTV *env*-like sequences on microdissected FFPE sections
Park, 2011 [112]	0/42 (0%)	Nested-PCR for MMTV-like *env* sequences on FFPE sections
Lawson, 2010 [113]	33/74 (54%) by PCR 5/27 (19%) by IS-PCR	IS-PCR and IHC for the gp52 *env* protein on FFPE sections
Fukuoka, 2008 [114]	0/46 (0%)	PCR for MMTV *env* sequences and Southern blot hybridization on CPT
Hachana, 2008 [115]	17/122 (14%)	Nested-PCR for MMTV *env* like on CPT

FFPE, formalin-fixed, paraffin-embedded; qPCR, quantitative PCR; RT-PCR, real-time PCR; CPT, cryopreserved tissue; IS-PCR, in situ PCR; IHC, immunohistochemistry.

**Table 4:** Summary of the prevalence of MMTV detection of investigated cases and controls.

Study	MMTV-Positive Cases/Total	MMTV-Positive Controls/Total	Statistically Significant? Y/N (*p*-Value)
Khalid, 2021 [99]	69/105 (65%)	2/15 (13%) ^§^	Yes (*p* = 0.0001)
De Sousa Pereira, 2020 [100]	41/217 (19%)	30/196 (15%) ^§^	No (*p* = 0.3624)
Seo, 2019 [102]	12/128 (9%)	0/128 (0%) ^§^	Yes (*p* = 0.0004)
Al Dossary, 2018 [103]	9/103 (9%)	0/51 *^,#,&^	Yes (*p* = 0.0301)
Shariatpanahi, 2017 [105]	19/59 (32%)	3/59 (5%) ^#^	Yes (*p* < 0.001)
Reza, 2015 [106]	12/100 (12%)	0/100 (0%) ^§^	Yes (*p* = 0.0003)
Ahangar Oskouee, 2014 [107]	0/65 (0%)	0/65 ^#^	//
Morales-Sánchez, 2013 [109]	0/86 (0%)	0/65 (0%) ^§^	//
Glenn, 2012 [17]	39/50 (78%) with liquid PCR 5/27 (19%) with IS-PCR	13/40 (32%) ^^^ with liquid PCR 3/18 (17%) ^^^ with IS-PCR	Yes, with liquid PCR (*p* = 0.0001) No with IS-PCR (*p* = 1.0000)
Lawson, 2010 [113]	33/74 (54%) with liquid PCR 5/27 (19%) by IS-PCR	0/29 (0%) ^§^ with liquid PCR 3/18 (17%) ^§^ with IS-PCR	Yes with liquid PCR (*p* = 0.0001) No with IS-PCR (*p* = 1.0000)
Hachana, 2008 [115]	17/122 (14%)	0/122 (0%) ^§^	Yes (*p* < 0.0001)

* fibroadenomas, ^#^ unspecified benign lesions, ^§^ normal breast tissue from the same patient, ^^^ normal breast tissue from healthy patients, ^&^ fibrocystic disorder, //, data not available.

**Table 5:** Prevalence of EBV detection in breast cancer samples according to different studies.

Study	Positive Cases/Total Cases (%)	Viral Proteins Detected	Technique Used
Gouadfel, 2023 [128]	7/30 (23%)	LMP1	IHC on FFPE sections
Mekrazi, 2023 [129]	0/100 (0%) with IHC 44/100 (44%) with PCR 0/15 (0%) with ISH	//	IHC, PCR, ISH on FFPE sections
Gupta, 2022 [25]	36/74 (49%)	//	PCR on FFPE sections
Oliveira, 2022 [27]	5/72 (7%) with ISH 50/72 (69%) with IHC	EBNA1, EBER1	ISH and IHC on FFPE sections
Shahi, 2022 [130]	25/130 (19%)	//	PCR and IHC on FFPE
Zhang, 2022 [131]	57/140 (41%)	//	CISH on FFPE sections
Ghaffari, 2021 [132]	12/72 (17%)	//	PCR on FFPE sections
Gupta, 2021 [31]	25/70 (36%)	EBNA1 (55/70), EBNA2 (31/70)	PCR on FFPE sections
Charostad, 2021 [133]	6/51 (12%)	//	PCR on CPT
Golrokh Mofrad, 2020 [134]	4/59 (7%)	EBNA 1	PCR on FFPE
Mostafaei, 2020 [135]	38/83 (46%)	//	PCR on unspecified tissue sample
Dowran, 2019 [136]	0/150 (0%)	//	PCR on FFPE sections
Sharifpour, 2019 [137]	10/37 (27%)	EBNA3C	PCR on FFPE sections
Pai, 2018 [138]	25/83 (30%)	//	ISH on FFPE slides
El-Naby, 2017 [139]	10/42 (24%)	EBNA1, LMP1	Nested-PCR on IHC on FFPE slides
Naushad, 2017 [45]	83/250 (29%)	EBNA2	PCR on FFPE sections
Aboulkassim, 2015 [140]	56/108 (52%)	LMP1 EBNA1	PCR on TMA
Ballard, 2015 [141]	63/160 (39%)	//	IHC on TMA
Fimereli, 2015 [54]	1/58 (1%) with transcriptome, 0/58 (0%) with IHC		RNA sequencing, exome sequencing PCR and IHC on CPT and FFPE sections
Reza, 2015 [106]	8/100 (8%) with rt-PCR, 18/100 (18%) EBER RNA	//	RT-PCR
Richardson, 2015 [142]	24/70 (34%)	//	qPCR on CPT
Corbex, 2014 [61]	10/123 (8%)	EBV1 gene	PCR on FFPE sections
Peng, 2014 [63]	60/100 (60%)	//	Multiplex PCR detected by matrix-assisted laser desorption ionization-time of flight mass spectrometry on CPT
Marrão, 2014 [143]	22/85 (26%)	//	RT-quantitative light cycler PCR
Morales-Sánchez, 2013 [109]	4/86 (5%)	//	Nested-PCR on CPT
Baltzell, 2012 [144]	2/70 (3%) with PCR, 0/70 (0%) with ISH	//	ISH and IS-PCR on FFPE
Glenn, 2012 [17]	34/50 (68%) with liquid PCR, 5/27 (19%) with IS-PCR	EBNA1	Liquid PCR on CPT, IS-PCR on FFPE sections
Zekri, 2012 [145]	32/90 (35%)	EBNA1 EBER1	PCR and ISH on FFPE slides
Aguayo, 2011 [73]	3/46 (7%)	//	RT-PCR and ISH on FFPE sections
Hachana, 2011 [146]	33/123 (27%) with PCR 0/123 (0%) with ISH 0/123 (0%) with IHC	//	PCR, ISH and IHC on FFPE sections
Kadivar, 2011 [147]	0/100 (0%)	//	IHC and PCR on FFPE sections
Mazouni, 2011 [148]	65/196 (33%)	//	RT-PCR on CPT
Lorenzetti, 2010 [127]	22/71 (31%)	//	ISH and PCR on FFPE
Joshi, 2009 [149]	28/58 (55%)	EBNA 1	IHC on FFPE slides
Fawzy, 2008 [150]	10/40 (25%)	//	IHC on FFPE sections

IHC, immunohistochemistry; FFPE, formalin-fixed, paraffin-embedded; //, data not available; ISH, in situ hybridization; CISH, chromogenic in situ hybridization; TMA, tissue microarray; CPT, cryopreserved tissue; RT-PCR, real-time PCR; qPCR, quantitative PCR; IS-PCR, in situ PCR.

**Table 6:** Summary of the prevalence of EBV detection of investigated cases and controls.

Study	EBV-Positive Cases/Total	EBV-Positive Controls/Total	Statistically Significant? Y/N (*p*-Value)
Charostad, 2021 [133]	6/51 (12%)	1/51 (1%) ^§^	No (*p* = 0.0599)
Golrokh Mofrad, 2020 [134]	4/59 (7%)	0/59 (0%) ^#^	No (*p* = 0.1186)
Mostafaei, 2020 [135]	38/83 (46%)	5/31 (16%) ^#^	Yes (*p* = 0.004)
Dowran, 2019 [136]	0/150 (0%)	0/150 (0%) *^,&^	//
Sharifpour, 2019 [137]	10/37 (27%)	4/35 (18%) *	No (*p* = 0.09)
El-Naby, 2017 [139]	10/42 (24%)	6/42 (14%) *	No (*p* = 0.4)
Reza, 2015 [106]	8/100 (8%) with Real time PCR 18/100 (18%) with Eber RNA	0/100 (0%) ^§^	Yes (*p* = 0.0068)
Richardson, 2015 [142]	24/70 (34%)	9/70 (12%) ^§^	Yes (*p* = 0.0048)
Peng, 2014 [63]	60/100 (60%)	16/50 (32%) ^#^	Yes (*p* = 0.0017)
Glenn, 2012 [17]	34/50 (68%) with liquid PCR, 5/27 (19%) with IS-PCR	14/40 (35%) ^^^ with liquid PCR, 6/18 (33%) ^^^ with IS-PCR	Yes with liquid PCR (*p* = 0.0028) No with IS-PCR (*p* = 0.3040)
Zekri, 2012 [145]	32/90 (35%)	0/20 (0%) ^^^	Yes (*p* = 0.0007)
Hachana, 2011 [146]	33/123 (27%)	0/123 (0%) ^§^	Yes (*p* < 0.0001)
Kadivar, 2011 [147]	0/100 (0%)	0/42 (0%) ^#^	//
Lorenzetti, 2010 [127]	22/71 (31%)	0/48 (0%) ^#^	Yes (*p* < 0.0001)
Joshi, 2009 [149]	28/58 (55%)	0/30 (0%) ^#^	Yes (*p* < 0.0001)
Fawzy, 2008 [150]	10/40 (25%)	0/10 (0%) ^&^	No (*p* = 0.1791)

* fibroadenomas, ^#^ unspecified benign lesions, ^§^ normal breast tissue from the same patient, ^^^ normal breast tissue from healthy patients, ^&^ fibrocystic disorder, //, data not available.

**Table 7:** Prevalence of CMV detection in breast cancer samples according to different studies.

Study	Positive Cases/Total Cases (%)	Technique Used
Touma, 2023 [162]	9/109 (8%) for IE protein, 82/108 (76%) for LA protein	IHC on FFPE TMA
Calderon, 2022 [24]	169/233 (73%)	IHC on FFPE sections
Ghaffari, 2021 [132]	5/72 (7%)	Nested-PCR on FFPE sections
Golrokh Mofrad, 2021 [30]	0/59 (0%)	Nested-PCR on FFPE sections
Nakhaie, 2021 [163]	8/49 (16%)	PCR on CPT
Touma, 2021 [164]	83/111 (75%) for LA protein, 9/111 (8%) for IE protein	IHC on FFPE sections
Costa, 2019 [165]	49/49 (100%) for IE protein, 11/49 (22%) for LA protein	PCR on FFPE sections
Sepahvand, 2019 [166]	20/37 (54%)	PCR on FFPE sections
El Shazly, 2018 [167]	11/61 (18%) for CMV DNA 21/61 (34%) for IE protein	PCR on FFPE sections
Richardson, 2015 [142]	0/70 (0%)	qPCR on CPT
Bakhtiyrizadeh, 2017 [42]	0/150 (0%)	RT-PCR on FFPE sections
El-Shinawi, 2013 [168]	62%	Nested-PCR on FFPE sections

IHC, immunohistochemistry; FFPE, formalin-fixed, paraffin-embedded; TMA, tissue microarray; CPT, cryopreserved tissue; qPCR, quantitative PCR; RT-PCR, real-time PCR.

**Table 8:** Summary of the prevalence of CMV detection of investigated cases and controls.

Study	CMV-Positive Cases/Total	CMV-Positive Controls/Total	Statistically Significant? Y/N (*p*-Value)
Nakhaie, 2021 [163]	8/49 (16%)	1/49 (2%) ^§^	Yes (*p* = 0.0307)
Costa, 2019 [165]	49/49 (100%) for IE protein, 11/49 (22%) for LA protein	2/26 (8%) ^§^ for IE protein 0/26 (0%) ^§^ for LA protein	Yes (*p* = 0.0001) for IE protein, (*p* = 0.0126) for LA protein
Sepahvand, 2019 [166]	20/37 (54%)	10/35 (26%) *	Yes (*p* = 0.0340)
El Shazly, 2018 [167]	11/61 (18%)	1/20 (5%) *	No (*p* = 0.2765)
Bakhtiyrizadeh, 2017 [42]	0/150 (0%)	2/150 (1%) ^#^	No (*p* = 0.4983)
Richardson, 2015 [142]	0/70 (0%)	2/70 (3%) ^§^	No (*p* = 0.4964)

* fibroadenomas, ^#^ unspecified benign lesions, ^§^ normal breast tissue from the same patient.

**Table 9:** Prevalence of BLV detection in breast cancer samples according to different studies.

Study	Positive Cases/Total Cases (%)	Technique Used
Amato, 2023 [171]	0/30 (0%)	PCR on CPT
Yamanaka, 2022 [172]	0/23 (0%)	PCR on CPT
Adekanmbi, 2021 [173]	0/238 (0%)	Fluorescence resonance energy transfer-qPCR on blood-derived DNA samples
Olaya-Galán, 2021 [174]	46/75 (61%)	Nested-liquid phase PCR on FFPE sections
Delarmelina, 2020 [175]	47/49 (96%) 46/49 (94%) for the *tax* gene 28/49 (57%) for the *env* gene	Nested-PCR on FFPE
Schwingel, 2019 [176]	22/72 (31%)	PCR on FFPE sections
Baltzell, 2018 [36]	35/61 (57%)	PCR and/or DNA hybridization on FFPE sections
Buehring, 2017 [177]	40/50 (80%)	PCR-ISH on FFPE sections
Gillet, 2016 [178]	0/51 (0%)	Raw DNA sequences from whole genomes of breast tumors and normal breast tissues adjacent to the tumor were retrieved from the NCBI database of genotype and phenotype.
Zhang, 2016 [179]	0/91 (0%)	RT-PCR on unspecified tissue samples
Buehring, 2015 [180]	67/114 (59%)	IS-PCR on FFPE sections
Buehring, 2014 [181]	97/2019 (44%)	IS-PCR on FFPE sections

CPT, cryopreserved tissue; qPCR, quantitative PCR; FFPE, formalin-fixed, paraffin-embedded; PCR-ISH, PCR-in situ hybridization; RT-PCR, real-time PCR; IS-PCR, in situ PCR.

**Table 10:** Summary of the prevalence of BLV detection of investigated cases and controls.

Study	BLV-Positive Cases/Total	BLV-Positive Controls/Total	Statistically Significant? Y/N (*p*-Value)
Olaya-Galán, 2021 [174]	46/75 (61%)	40/83 (48%) ^#^	No (*p* = 0.1113)
Delarmelina, 2020 [175]	47/49 (96%) 46/49 (94%) for the *tax* gene 28/49 (57%) for the *env* gene	23/39 (59%) ^^^ 20/39 (51%) ^^^ for the *tax* gene 14/39 (36%) ^^^ for the *env* gene	Yes (*p* < 0.001)
Schwingel, 2019 [176]	22/72 (31%)	10/72 (14%) ^^^	Yes (*p* = 0.0265)
Baltzell, 2018 [36]	35/61 (57%)	20/103 (20%) ^§^	Yes (*p* < 0.0001)
Buehring, 2017 [177]	40/50 (80%)	19/46 (41%) ^#^	Yes (*p* = 0.0001)
Buehring, 2015 [180]	67/114 (59%)	30/104 (29%) ^^^	Yes (*p* < 0.0001)

^#^ unspecified benign lesions, ^§^ normal breast tissue from the same patient, ^^^ normal breast tissue from healthy patients.

**Table 11:** Prevalence of MCPyV detection in breast cancer samples according to different studies.

Study	Positive Cases/Total Cases (%)	Technique Used
Reza, 2015 [106]	3/100 (3%)	RT-PCR on FFPE sections
Corbex, 2014 [61]	1/123 (1%)	PCR on FFPE sections
Peng, 2014 [63]	14/100 (14%)	Multiplex PCR detected by matrix-assisted laser desorptionionization-time of flight mass spectrometry on CPT
Khan, 2012 [189]	0/58 (0%)	PCR on FFPE sections

RT-PCR, real-time PCR; FFPE, formalin-fixed, paraffin-embedded; CPT, cryopreserved tissue.

## Data Availability

The original contributions presented in this study are included in the article. Further inquiries can be directed to the corresponding author.
